# Compliance with Guideline-Directed Medical Therapy and Early Implantable Cardioverter-Defibrillator Activation in Heart Failure: A Retrospective Study

**DOI:** 10.31083/j.rcm2503075

**Published:** 2024-02-27

**Authors:** Ivan Prepolec, Vedran Pašara, Andrija Nekić, Jakov Emanuel Bogdanić, Jurica Putrić Posavec, Borka Pezo Nikolić, Miroslav Krpan, Richard Matasić, Mislav Puljević, Martina Lovrić Benčić, Davor Puljević, Davor Miličić, Carlo de Asmundis, Gian Battista Chierchia, Giacomo Mugnai, Vedran Velagić

**Affiliations:** ^1^Department of Cardiovascular Diseases, University Hospital Centre Zagreb, 10000 Zagreb, Croatia; ^2^University of Zagreb School of Medicine, 10000 Zagreb, Croatia; ^3^Vrije Universiteit Brussel, 1050 Brussel, Belgium; ^4^Azienda Ospedaliera Universitaria Integrata Verona, 37126 Verona, Italy

**Keywords:** heart failure, implantable cardioverter-defibrillator, guideline-directed medical therapy, compliance

## Abstract

**Background:**

This study was conducted to evaluate compliance with guideline-directed optimal medical therapy (OMT) and its association with early implantable cardioverter-defibrillator (ICD) activation in patients with heart failure and reduced ejection fraction (HFrEF).

**Methods:**

Retrospective data from 307 patients who underwent ICD implantation for primary prevention from 2011 to 2017 were collected and analyzed.

**Results:**

Among the study participants, only 23.8% received the maximum tolerated dose of OMT prior to ICD implantation, with 59.0% receiving all three OMT medication groups. No significant difference in OMT compliance was found between patients with ischemic cardiomyopathy (ICM) and those with non-ischemic dilated cardiomyopathy (DCM). However, DCM patients received ICDs more frequently at the time of diagnosis than ICM patients (13.8% vs. 0.7%). Early ICD activation (within 3 months) occurred in only one patient who had not received appropriate OMT, representing 0.7% of all ICM patients. Furthermore, early activation was also infrequent in patients who received OMT (2.9% of ICM patients and 2.6% of DCM patients). Echocardiography follow-up data revealed that 20.4% of ICM patients and 29.8% of DCM patients who did not receive OMT before ICD implantation showed improvement in the left ventricular ejection fraction (EF) to 35% or more.

**Conclusions:**

This study found suboptimal compliance with OMT prior to ICD implantation in HFrEF patients. The results showed that early ICD activation was rare in all patient groups, especially those who did not receive the prescribed 3 months of OMT. More research is needed to investigate longer waiting periods for the evaluation of potential EF improvement, and to better evaluate the eligibility of HFrEF patients for ICD. The current findings have potential implications for clinical practice and patient outcomes.

## 1. Introduction

Implantable cardioverter-defibrillators (ICDs) are the gold standard therapy for primary prevention of sudden cardiac death (SCD) in patients with heart failure with reduced ejection fraction (HFrEF). The evolution of medical therapy for heart failure has led to a progressive change in the guidelines. According to the current European Society of Cardiology (ESC) guidelines, ICD implantation is indicated only after patients have been treated with optimal medical treatment (OMT) for three months [1]. However, real-world data shows that many patients are still undertreated before ICD implantation [2, 3]. This is important since OMT can improve the ejection fraction (EF), especially in patients with newly diagnosed non-ischemic dilated cardiomyopathy (DCM) [4]. Although some patients may no longer be candidates for ICD following improvement in EF, the evidence suggests they could still be at higher risk of arrhythmia [5, 6]. Trials with wearable cardioverter-defibrillators (WCD) indicate that patients may be at risk early after diagnosis when medical therapy is being up-titrated [7, 8, 9, 10]. More data from registries and additional randomized trials are needed to clarify these important issues and to help identify patients who will benefit most from ICD therapy as primary prevention.

The aim of this study was to investigate compliance with current guideline-directed OMT in patients with HFrEF during the 3-month period prior to ICD implantation for the primary prevention of SCD [1]. We also evaluated the rate of early ICD activation during the first 3 months (OMT introduction and titration period) after implantation in patients who did not receive OMT, or had ICD implanted soon after revascularization procedures. When available, echocardiography data was analyzed to assess the improvement in EF following OMT.

## 2. Materials and Methods

Data were retrospectively collected for all patients with HFrEF who were newly implanted with an ICD for the primary prevention of SCD at the University Hospital Centre in Zagreb between January 2011 and December 2017. Patients already treated with cardiac resynchronization therapy (CRT) and ventricular assist devices (VAD) were excluded, as well as those with pre-existing ICDs who were admitted for box change. Therapy compliance with the guidelines was evaluated using hospital medical records. Patients treated with the maximum tolerable dose of angiotensin-converting-enzyme inhibitor (ACEI) or angiotensin II receptor blockers (ARB), beta-blocker (BB) and mineralocorticoid receptor antagonist (MRA) for 3 months before implantation were considered to have received OMT, as defined in the 2012 and 2016 ESC guidelines [11, 12]. The use of sacubitril/valsartan and sodium-glucose cotransporter 2 (SGLT2) inhibitors was not addressed here due to the study period being prior to their introduction in the guidelines. When medical therapy was started <3 months before the implant, or the dose of ACEI, BB or MRA was up-titrated during hospitalization for ICD implantation or during 1-year of follow-up, patients were considered not to have received OMT. Patients with contraindications or intolerance to BB, ACEI or MRA were considered as being treated according to the guidelines. Patients were classified into two groups to better assess possible differences in the approach of physicians to ICD implantation, and to assess the potential for improvement in cardiac function. These were the ischemic cardiomyopathy (ICM) group, and the DCM group. For ICM patients, the length of time since the last revascularization procedure was recorded. Patients were considered to be treated according to guidelines if the ICD was implanted at least 6 weeks after ST-elevation myocardial infarction (STEMI), or 3 months after percutaneous coronary intervention (PCI). Data was collected on ICD activation (shock or anti-tachycardia pacing, ATP) during the first 3 months after implantation. The aim was to assess the risk of SCD and the benefit of premature ICD implantation during the first 3 months when OMT was started and up-titrated. Follow-up echocardiography data was analyzed to determine whether cardiac function, as measured by left ventricular EF, improved to 35% or more at 6-months after ICD implantation.

This research was conducted in accordance with the principles of the Declaration of Helsinki and was approved by the Ethics committee of the University Hospital Center, Zagreb (class 8.1-23/218-2, number 02/013 AG).

Categorical variables are expressed as absolute and relative frequencies. Continuous variables showed normal distribution and were expressed as mean ± standard deviation. Comparisons of continuous variables were performed with Student’s *t* test, and binomial variables with the χ^2^ or Fisher’s exact test, as appropriate. A two-tailed probability value of <0.05 was deemed significant. Statistical analyses were conducted using SPSS software (t v22, IBM Corp., Chicago, IL, USA).

## 3. Results

A total of 307 ICDs were implanted during the study period, with 147 (47.9%) in ICM patients and 160 (52.1%) in DCM patients. Baseline characteristics for the two patient groups are shown in Table 1.

**Table 1.** **Patient characteristics**.

	ICM	DCM	*p*-value
Patients, n (%)	147 (47.9)	160 (52.1)	/
Male, n (%)	134 (91.2)	136 (85.0)	0.12
Age (years)	61.2 ± 9.2	54.1 ± 13.6	<0.01
LVEF, %	28.8 ± 7.0	26.7 ± 7.8	0.01
NYHA functional status	2.0 ± 0.7	2.1 ± 0.9	0.76
BB, %	96.6	91.9	0.09
ACEI or ARB, %	83.7	75.6	0.09
MRA, %	52.4	58.1	0.36
Recent revascularization, n (%)	9 (6.1)	/	/
Receiving OMT at implant, n (%)	34 (23.1)	39 (24.4)	0.89

ICM, ischemic cardiomyopathy; DCM, non-ischemic dilated cardiomyopathy; LVEF, left ventricular ejection fraction; BB, beta blocker; ACEI, angiotensin-converting-enzyme inhibitor; ARB, angiotensin II receptor blockers; MRA, mineralocorticoid receptor antagonist; recent revascularization = less than 3 months after revascularization procedure, or less than 6 weeks after ST-elevation myocardial infarction; OMT, optimal medical therapy; NYHA, New York Heart Association.

Only 37 patients (12.05%) were female, and mean age at implantation was 57.5 ± 12.2 years. The mean New York Heart Association (NYHA) functional status was similar in the ICM and DCM groups (2.1 ± 0.8), but left ventricular EF was slightly higher in the ICM group (28.8 ± 7.0 vs. 26.7 ± 7.8, *p* < 0.01). Only 23.1% of ICM patients were treated with the maximum tolerable dose of OMT at least 3 months before implantation, and a similar proportion of DCM patients (24.4%). In the overall patient cohort, 79.5% were receiving ACEI or ARB at the time of implantation, 94.1% were receiving BB, and 55.4% were receiving MRA at the time of implantation. No significant differences were observed between the two groups for the use of these drugs (Table 1). Only 57.8% of ICM patients and 60.0% of DCM patients were treated with all three medication groups before implantation. However, the dose of OMT was up-titrated during hospitalization for ICD implantation in 22.1% of cases (21.1% of ICM patients and 23.1% of DCM patients), or during the first year after implantation in 13.0% of cases (13.6% of ICM patients and 12.5% DCM patients). Importantly, ICDs were implanted at the time of diagnosis significantly more often in DCM patients than in ICM patients (13.8% vs. 0.7%, respectively, *p* < 0.01) (Table 2). In 9 (6.1%) ICM patients, the ICD was implanted less than 3 months after revascularization procedures (6 cases), or less than 6 weeks after STEMI (3 cases).

**Table 2.** **Compliance with guideline-directed OMT before ICD implantation**.

	ICM: OMT	ICM: no OMT	DCM: OMT	DCM: no OMT	*p*-value
Patients, n (%)	34 (23.1)	113 (76.9)	39 (24.4)	121 (75.6)	0.89
ICD implantation at diagnosis, n (%)	/	1 (0.9)	/	22 (18.2)	<0.01
OMT <3 months, n (%)	/	3 (2.7)	/	6 (5.0)	0.50
Incomplete OMT*, n (%)	/	58 (51.3)	/	36 (29.8)	<0.01
Dose up-titrated during hospitalization, n (%)	/	31 (21.1)	/	37 (23.1)	0.25
Dose up-titrated during 1 year follow-up, n (%)	/	20 (13.6)	/	20 (12.5)	0.86

DCM, non-ischemic dilated cardiomyopathy; ICM, ischemic cardiomyopathy; OMT, optimal medical therapy; ICD, implantable cardioverter-defibrillator. * incomplete OMT = at least one of the three drug groups (beta-blocker, angiotensin-converting-enzyme inhibitor or angiotensin II receptor blockers, mineralocorticoid receptor antagonist) was not given.

Among all patients who had not received OMT according to the guidelines and who prematurely received an ICD, follow-up data revealed early appropriate activation (within 3 months of implantation) in only one ICM patient (0.7% of ICM patients). The patient had established ICM and was suffering from recurrent, unexplained syncopes. No early ICD activations were observed in the DCM patients who had not received OMT before implantation. However, 4 patients from that group (3.3%) received inappropriate ICD shock due to supraventricular tachycardia or, in one case, lead dysfunction. Appropriate early ICD activations were uncommon even in patients who had received OMT, with only 1 (2.9%) ICM patient and 1 (2.6%) DCM patient receiving activation within the first 3 months of implantation (Table 3).

**Table 3.** **Early ICD activation and follow-up left ventricular systolic function**.

	ICM: OMT	ICM: no OMT	*p*-value	DCM: OMT	DCM: no OMT	*p*-value
Patients, n (%)	34 (23.1)	113 (76.9)	/	39 (24.4)	121 (75.6)	/
Appropriate activation in first 3 months, n (%)	1 (2.9)	1 (0.9)	0.41	1 (2.6)	0 (0)	0.24
Inappropriate shock in first 3 months, n (%)	0 (0)	0 (0)	1.00	1 (2.6)	4 (3.3)	1.00
EF improved to ≥35% after 1 year, n (%)	5 (14.7)	23 (20.4)	0.62	4 (10.3)	36 (29.8)	0.02

EF, ejection fraction; ICD, implantable cardioverter-defibrillator; OMT, optimal medical therapy; DCM, non-ischemic dilated cardiomyopathy; ICM, ischemic cardiomyopathy.

Follow-up echocardiography data during the first year after implantation was available in 55.4% of cases. In 23 (20.4%) ICM patients and 36 (29.8%) DCM patients who did not receive OMT prior to implantation, the EF improved to 35% or more during the first year after implantation. DCM patients who did not receive OMT before implantation were significantly more likely to improve than those with OMT (OR 3.71, 95% CI 1.23–11.2, *p* = 0.02). Some patients showed improvement after ICD implantation, even though they were already on the maximum tolerable dose of OMT beforehand. This occurred in 5 (3.4%) ICM patients and in 4 (2.5%) DCM patients (Table 3).

## 4. Discussion

The current results show that the rate of OMT before ICD implantation in HFrEF patients was less than optimal. Just 23.8% of patients received the maximum tolerable dose of OMT, while 59.0% received the three medication groups at any dose. It is important to emphasize that our results were obtained from a single-center, retrospective study, and that two larger registries reported an OMT rate of 61.1% and 73.5% [2, 3]. However, one of these only included patients who were older than 65 years [2]. Furthermore, these registries were established during the era when OMT was considered to be ACEI and BB. In addition to the inclusion of MRA, another key difference with the present study is that we obtained our data directly from hospital medical records rather than from insurance databases or prescription fills. This allowed us to track changes in the dose of OMT and to better assess compliance. As mentioned above, the guidelines were changed during the study period. According to the 2006 American College of Cardiology (AHC)/American Heart Association (AHA)/ESC guidelines, “chronic optimal medical therapy” was required before ICD implantation [13]. At the time, MRA was not required and the duration of therapy was not specified [14]. Guidelines for the treatment of heart failure (HF) patients were subsequently updated [11, 15], but only in 2015 did the ESC guidelines adopt the current recommendation of at least 3 months of treatment with maximum tolerable doses of ACEI, BB and MRA before ICD implantation [16]. This is not surprising, since a considerable proportion of patients in the landmark ICD trials were not treated with ACEI or BB [17, 18].

Given the advances in pharmacological treatment of HF with newly developed drugs (sacubitril/valsartan, sodium-glucose cotransporter 2 inhibitors, ivabradine, vericiguat and omecamtiv mecarbil), the introduction and up-titration of medical therapy now takes longer. It has been suggested this might justify the need for a longer waiting period of up to 6 months before ICD implantation in DCM cases [19]. This is also supported by the results of two studies showing at least 20% improvement in EF in 39% of DCM patients after 6-months treatment with ACEI and BB [20]. Indeed, a significant proportion of cases no longer met the criteria for an ICD [21]. The available echocardiography data from our registry is in line with these findings. The EF improved to >35% in 20.4% of ICM patients and 29.8% of DCM patients who were not receiving OMT at the time of implantation. It is worth repeating that the majority of patients in our study (76.9% ICM and 75.6% DCM) were not receiving appropriate OMT at the time of implantation. Some patients who received OMT also improved, but DCM patients who were already receiving OMT were significantly less likely to improve than those without OMT at implantation (10.3% vs. 29.8%, OR 3.7, 95% CI 1.2–11.2). This may be because some OMT-treated DCM patients were not considered for ICD implantation because they had already improved with appropriate OMT. Overall, EF improved in 19% of ICM patients and in 25% of DCM patients. This finding is similar to another study which showed that EF improved to >35% in 24% of OMT-treated DCM patients at 12 months follow-up after ICD implantation [4]. Predicting which patients might improve remains challenging, especially in the context of ICM where myocardial viability can be helpful but is not always associated with reduced mortality [22]. A recent magnetic resonance imaging (MRI) study of DCM patients found that a very low baseline EF or a high QRISK3 score reduced the possibility of left ventricular recovery [23]. Differentiation between ICM and DCM patients is also supported by current ESC guidelines [1] that lower the level of recommendation for primary prophylaxis ICD implantation in DCM patients. It is also supported by a Danish study that showed no reduction in all-cause mortality in DCM patients over the age of 70 years [24].

A previous study found that the rate of appropriate ICD activation was similar in all patients during the first year, but subsequently decreased in patients whose EF improved compared to those whose EF remained low [4]. Smer *et al*. [5] and Pillarisetti *et al*. [6] both reported that the risk of arrhythmia remains elevated even after EF improves, although it is lower compared to patients with no improvement in EF. HFrEF patients are perceived to be at high risk of SCD, and therefore it is interesting that we observed a very low rate of appropriate ICD activation. Only 3 (1.0%) patients with appropriate activation were observed during the first 3 months after implantation (1 no-OMT ICM patient and 2 OMT patients), with no deaths during this period. This is lower than the rate reported in WCD trials of patients with newly diagnosed ICM or DCM, which ranged from 1.4% to 7.1% [7, 8, 9, 10]. However, there are no randomized trials examining WCD for newly-diagnosed cardiomyopathy during the introduction and up-titration of OMT. It is also important to consider the burden of inappropriate shocks on DCM patients observed during the first 3 months in our study (2.6% of OMT and 3.3% of non-OMT DCM patients). This has a negative impact on quality of life [25] and represents a significant proportion of patients compared to the 1-year incidence of 7% reported elsewhere [26].

The available data and the present research findings support the current practice of a 3-month “waiting period”. This helps to avoid a significant number of unnecessary implantations. An even longer waiting period of 6-months could allow enough time for positive remodeling in patients with DCM, prior to re-evaluation for ICD implantation [18]. This could also be supported by the very low incidence of early ICD activation in the present study, and the declining risk of SCD in HF patients reported in a large meta-analysis [27], even before introduction of the current quadruple OMT. However, some patients remain at high risk of SCD despite EF improvement. The use of other criteria to identify these patients is currently being investigated, including late gadolinium enhancement on cardiac magnetic resonance [28].

## 5. Conclusions

Analysis of our institutional ICD registry revealed that a significant number of patients with HFrEF received premature ICD implants before appropriate treatment with OMT. This was more common in patients with newly diagnosed DCM. Our follow-up data suggests that early ICD activation is very rare in all cases and hardly ever happens in patients who received an ICD immediately after diagnosis, revascularization, or without receiving 3 months of OMT. The rate of inappropriate shocks in DCM patients is non-negligible, even early after implantation. A significant percentage of patients improve after appropriate medical treatment. The present findings support current guidelines, but also suggest the possibility of longer waiting periods for titration of OMT before ICD implantation, especially in DCM patients. There were several important limitations to this study, including its single center design, and the use of older OMT regimens without SGLT2 inhibitors. Additional research would add significantly to our understanding of the role and importance of OMT before ICD implantation in HFrEF patients.

## Data Availability

In compliance with the data sharing policy, we regret that the dataset associated with this study is unavailable to the public due to restrictions imposed by the data custodian, as access is limited to authorized individuals with specific permissions for data security and privacy reasons.
